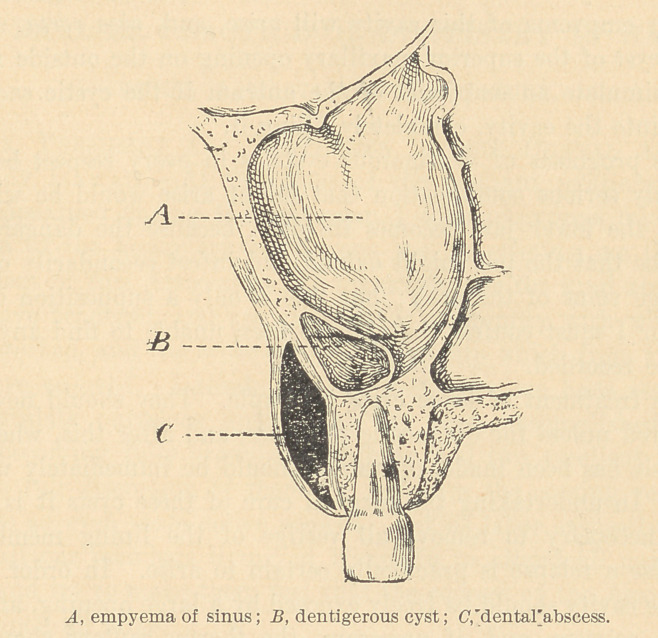# Dentigerous Cysts

**Published:** 1904-04

**Authors:** Charles Greene Cumston

**Affiliations:** Boston, Mass.


### DENTIGEROUS CYSTS.[1]

BY CHARLES GREENE CUMSTON, M.D., BOSTON, MASS.

In considering the subject of dentigerous cysts, a few remarks on the pathology of the condition may not be out of place, as the literature is rather scarce relative to the condition under consideration. The first important work on the subject was published in 1872 from the pen of the well-known Parisian dentist, Magitot. After a careful study of all material he had at hand, Magitot gave a systematic classification of dental cysts, and at the same time he took their etiology into consideration.

As most of the writers who have studied this question since Magitot's paper appeared have concurred in most of the statements therein found, it would seem proper to consider his paper a little in detail. Magitot considered every cystic production lined with a membrane and situated in the interior of the jaws as a maxillary cyst. These cysts usually contain fluid contents varying in nature from one case to another; they may be thin and fluid or, on the other hand, quite thick and pasty. Every cystic production arising spontaneously in the maxilla usually originates from a tooth. Magitot also believed that if a foreign body penetrated the jaw a cyst could form around it, and this fact has been proved by a case long ago published by Maisonneuve.

Cysts developing spontaneously he termed progenous cysts, and, in contrast to these, those developing around a foreign body he termed perigenous cysts. To these two forms which develop in the bone substance he adds a third type, to which he applied the name of neogenous cysts, which develop outside the osseous tissue.

Magitot subdivided follicular cysts into embryoplastic, odontoplastic, and corona-forming-period cysts according to the time of their development, and he again subdivides them according to the nature of their contents into serous, colloid, and caseous cysts.

By the classification given, it is readily seen how Magitot accounts for the formation of progenous cysts. From some influence he believes that the enamel may perish in different stages of development by either resorption or by maceration. The dental sac then

---

[1] Read by invitation before the Harvard Odontological Society, October 29, 1903.

slowly develops by a proliferation of its structures. The contents found in embryoplastic cysts will be composed of shapeless embryonal dental elements, in those of the odontoplastic cysts more or less regularly developed tooth elements will be present, while in cysts arising during a later period of dental development coronal structures should be present.

Magitot believed that neogenous cysts arise in the periosteum of the root, and he supposes that this membrane undergoes development by proliferation of its cellular structures and thus forms a cystic sac. The primary causative factor is an obliteration of the canal of the root due to an inflammatory thickening of the periosteum following infection from the canal of the root or by formation of a denticle in the pulp. The epithelial lining of these cysts is derived from a marked proliferation of the connective tissue cells.

Although Magitot unquestionably did much to enlighten us on the question, he could not reconcile his theory with facts,—namely, that the epithelium could arise as if by regeneration from the connective tissue elements. It was naturally most important to discover the true origin of the epithelium which is exclusively found in neogenous cysts when it has not become destroyed by a suppurative process of the contents of the cyst.

In 1885 appeared a paper by Malassez, and it is to him we are indebted for discovering in the maxillæ of embryos in different stages of development that during the period of the formation of the teeth there takes place, besides the physiological proliferation of the epithelium for the formation of the dental follicle from the embryonal enamel, other proliferative processes, arising both from the organon adamantinæ and from the mucous membrane. These epithelial masses are in structure exactly like the enamel organ, arranged around the root of the tooth in various manners. They are also found present in normal lower jaws of adults, and he came to the conclusion that these epithelial masses persist not only during the period of dentition, but are normally present during adult life. He asserted that not only the neogenous cysts of Magitot, but also every other neoplasm having an epithelial character developing in the jaw, especially multilocular cystomata and cystadenomata of the maxilla, originate from these masses of cells which he terms paradental epithelial débris.

The conclusions arrived at by Malassez were examined and found correct by other investigators. Von Brunn, especially, has given a satisfactory explanation, basing his assertion on the writings of Hartwig, published in 1874, and he shows how the epithelial masses, pre-existing around the root of the tooth, become constricted by the fetal enamel organ, and from its connective tissue with the epithelium of the buccal mucous membrane. If Malassez's theory relative to the origin of periodontal cysts from the paradental cell agglomeration is correct, these cysts should occasionally be met with in their early stages, and, in point of fact, Witzell published a paper on cysts of the roots of the teeth in 1896, in which he fully describes them and gives a satisfactory explanation for the development of periodontal cysts. He points out that one occasionally finds tumors varying in size from the head of a pin to a pea in extracted teeth, especially when they are the seat of caries. These neoplasms are sometimes spherical in shape, at others egg-shaped, varying in their connection with the roots, sometimes being near and sometimes being distant, while occasionally they are pedunculated.

On section these small, grayish-white bodies will be found composed of one or more cavities containing serous contents. Microscopically the external layer of these minute growths is composed of a rough connective tissue, then by a layer of tissue abounding in blood-vessels and leucocytes, while the cavities are lined with epithelium. The larger cystic cavities show a more evenly distributed epithelial layer, while in the smaller ones the thickness of the lining epithelium varies considerably.

We consequently are here dealing with a miniature cyst which on account of its diminutive size has never given rise to symptoms. The microscopical examination of well-developed cysts exactly corresponds with these small pathologic productions described by Witzel.

In the first case of dental cyst that I have to report, the lining membrane consisted of several layers of flattened epithelium, and outside of this was a connective tissue layer measuring about 1½ millimetres in thickness. Outside of this connective tissue layer was found another of connective tissue in which nuclei were easily distinguished. The history of the patient was briefly as follows: She was a well-built girl of twenty-five, who had always enjoyed good health. About three years ago she noticed for the first time a small bunch under the left nostril. This small growth gave rise to no pain and grew slowly, and continued to do so until, at the time of the patient coming under observation, the left nasolabial fold had completely disappeared, while a tumor about the size of a large walnut was found immediately underneath the labionasal fold. The skin covering the growth was movable, while the left nostril was pushed upward. By palpation, fluctuation could be detected.

Under ether narcosis the growth was incised, giving issue to a transparent serum. The opening was enlarged, and it was then found that a cavity existed between the nasal cavity and the antrum of Highmore, into which the apex of the root of a small incisor was found protruding. After extraction of the tooth and the removal of a corresponding part of the alveolar process, the cavity was plugged with gauze and the skin incision united by wire sutures. These were removed on the fifth day, and the patient was discharged well in three weeks.

It is easy to understand why we have a flattened epithelium in large cysts, while in the smaller ones cubic epithelium is present because it is well known with what ease epithelial cells adapt themselves to the amount of pressure brought to bear on them. In all the cases of dentigerous cysts which have come under our observation, the microscope has revealed practically the same histological structure, but in some instances the epithelial layers were so thick that the lining membrane of the cyst was readily peeled out from the cavity. Such was the condition found in the two following cases:

A healthy boy ten years of age had had what was called by the mother a swollen cheek for over three years. The swelling had apparently never given rise to any pain. It had slowly increased until at the time the boy came under observation the deformity was very marked. By palpation, a tumor the consistency of bone was found in the anterior aspect of the right superior maxillary bone. The nasal cavities were normal. Under ether an incision was made over the most promintent part of the growth, the gum was stripped back, and the bone opened with the chisel. This led into a large cavity lined by a whitish membrane, which was easily stripped off and removed. The cavity contained a light-yellow serum, but no evidence of any tooth could be found within it. On account of the large size of the cavity, which might be estimated that of a small hen's egg, it was deemed more prudent to obtain drainage through the nose, so a communication was made with the right nostril and a drainage-tube inserted. In terminating the operation, a portion of the anterior wall of the jaws was removed so that the cavity might contract more rapidly. Drainage was continued for a week, after which time the wound was found to be granulating nicely, and the patient was discharged cured at the end of four weeks.

The next case was a man thirty-seven years old, who for about eighteen months had noticed the presence of a tumor situated in the neighborhood of the left wisdom-tooth. The swelling was hard and had slowly increased in size. By examination an oval tumor was found situated in the neighborhood of the wisdom-tooth on the left-hand side, and might have measured in size that of a large English walnut. Under ether the last molar on the left was removed, and it was then found that we were dealing with a rather thick-walled cyst of the jaw. The cavity was split open with the chisel, and by exploration it was found to communicate with the antrum of Highmore. It was lined with a thick, whitish membrane, which was easily removed by blunt dissection. Drainage was obtained by making an opening into the nasal cavity, and a rubber tube was inserted, the cystic cavity being plugged with gauze. No evidence of a rudimentary tooth could be detected within the cavity. The liquid contents consisted of a thick, light-yellow serum.

Now, if we assume that these periodontal cysts are produced by an irritative process arising from the paradental epithelial débris of Malassez, and that the cavities increase in size on account of an increase of their fluid contents, we have all the conditions found in cysts of long standing explained. A cyst will naturally grow in the direction of the least resistance, and since the root of the tooth is firmly lodged in the alveolar process by its periosteum, the bone, being less resistant, gives way to the pressure. On account of the pressure on the surrounding structures produced by the growing cysts, the vessels and nerve supplying the tooth and periosteum of the root undergo pressure atrophy and resorption. Thus may be explained the absence of periosteum covering the root and the apices projecting into the cavity of the cyst, although their presence is by no means constant.

The perigenous cysts of Magitot can be explained by a foreign body directly in contact with, or in the neighborhood of, the so-called epithelial streaks of Brunn. This produces a constant irritation, which finally causes a proliferation of the latter, and to this type those cysts, in which fully developed teeth are found present, belong in all probability.

It is easily conceivable that a tooth which did not make its exit from the jaw from faulty position or some other similar circumstance becomes enclosed in the maxilla and at length plays the part of a foreign body, causing the epithelial streak to proliferate. It would appear that such cysts are generally considered under the head of follicular cysts, but under these circumstances it is difficult to explain the presence of fluid. But if we adopt Malassez's theory, it will be found to explain the formation of these cysts very well. One such case has come under my notice, the history of which is briefly as follows:

A woman sixty-four years old had had all her lower teeth removed several years ago. About twenty months before coming under observation a swelling made its appearance on the angle of the right jaw. The tumor gradually increased in size, so that when the patient was first seen it was as large as an egg. Later a certain amount of pain had been felt in the growth, and the latter had become quite tense and the patient complained of much annoyance from a throbbing sensation more or less constantly present. Under ether an incision was made through the skin over the most prominent part of the growth, and while peeling off the periosteum of the ascending ramus of the lower jaw a cystic cavity was opened, giving issue to a considerable quantity of malodorous pus. The cavity was at once enlarged, and by digital exploration the root of a tooth was found protruding into it. The root was extracted, and the cavity was thoroughly curetted and drained. The patient made an excellent recovery, although drainage was necessary for over three weeks on account of a more or less abundant discharge of pus, but she eventually recovered without a fistula.

If a tooth develops normally, it is evident that it can never become entirely enclosed within a cyst, but its root may be contained within the cavity, and this is what is found in the larger number of cases. Most of the text-books on surgery are very deficient in explaining the pathology of these cysts, and even commit gruesome errors in their statements. It is an unimpeachable fact that one tissue can only reproduce itself, and consequently epithelium can only be derived from epithelium; and it is also well known that the lining membrane of these cysts is always epithelial and never composed of granulation tissue. Now Malassez's theory explains satisfactorily the absence of the periosteal covering of the dental roots protruding into these cysts, but the cause giving rise to the proliferation of the epithelial streak of Brunn and to the formation of a periodontal cyst is not so easily demonstrated. Without any question an inflammation of the membrane of the root may be the starting-point of this process, and many cases unquestionably do originate in this way. In such cases caries of the tooth, inflammation or gangrene of the pulp must have preceded, but if the root of a perfectly normal tooth is found protruding into a cystic cavity, and where there is a complete absence of all signs of a periodontitis, or if no trace of tooth is found within the cavity of these cysts, such as has been observed, we consequently must admit that there are other causative factors existing. It is possible that traumatism may be the starting-point of some of these cysts, because the lower jaw is particularly well placed to receive external injuries, and it is a well-established fact that retained teeth may be the cause of periodontal cysts. But it is also well demonstrated that a perfectly normal tooth may be the etiological factor when such a tooth is deviated from its normal position, an example of which will be found in the following case:

A boy seventeen and a half years of age had always been fairly well excepting for the ordinary ailments of childhood. For the past eighteen months he had suffered more or less intensely from toothache. About five months ago he first noticed a slowly growing tumor on the left side of his cheek. The growth did not give rise to any pain. Examination showed that the tumor extended from the left nostril underneath the malar bone. The skin covering the growth was not reddened, and was found freely movable over the tumor. The teeth were in extremely bad condition. The left upper canine appeared healthy, but it pointed directly backward in an oblique direction. At this point the superior maxilla presents a swelling extending from the labial to the palatinal aspect. Crepitation could be elicited on pressure. Under ether an incision was made over the tumor, attacking it from inside the mouth, and when the cavity of the cyst was opened a large amount of thick, ill-smelling serum made its exit. The opening into the cyst was enlarged, the cavity being found lined with a smooth, glossy membrane. The apex of the root of the canine tooth was found protruding into the cystic cavity, and was extracted. The alveolar edge was chiselled away and the cavity packed with gauze and drained. The patient made a rapid and uneventful recovery. Examination of the tooth extracted showed that the apex of the root was bare of its periosteum.

On the other hand, the retention of developed teeth does not necessarily produce the formation of a cyst, as has been proved by several cases reported in literature. In one case reported by Hildebrand, that of a boy during his second dentition, he removed from the upper and lower jaws over two hundred perfectly developed teeth without any evidence of cystic formation around any of them.

It would also appear that the period of dentition may also play an important part in the etiology of periodontal cysts, and this should not seem surprising, because at that time processes of resorption and regeneration are taking place everywhere in the maxillæ. Now, during such a time of active metabolism there is no reason why an increased activity may not be present in the epithelial streak of Brunn.

In many cases periodontal cysts do make their début during the period of the second dentition, but in other cases the cysts begin to develop long before this period, as has been proved by certain cases reported in the literature. The second case narrated in this paper in all probability enters into this class, because evidently the cystic formation commenced at an early time in the dental development, and, in point of fact, microscopical examination showed a picture closely resembling an embryonal dentinal sac. This type of cyst may always be suspected if the tooth corresponding to it is absent, and this is an important point to be noticed in making a diagnosis. It may also occur that although the entire set of teeth is intact, a supernumerary tooth follicle may undergo cystic transformation, but such instances must be very rare. The cause of the cystic transformation is as yet unknown.

It may not be out of place to refer, in a few words, to a certain class of tumor which perhaps in reality does not strictly belong to the subject of this paper, but whose histological nature and origin is closely allied to that of periodontal cysts. The tumors to which I refer are usually encountered in the lower jaw near its angle. They suddenly begin to develop after they have been present for a number of years in a latent state, so to speak, and when they commence to increase in size they do so very markedly. Traumatism is usually the cause of their sudden increase in size and growth. After they have attained a certain size they present symptoms of cysts,—namely, fluctuation and parchment crepitation. These cysts have been treated by incision, curettage, and drainage, which has rarely been the means of curing them, and in most instances an extensive resection of the jaw has been necessary.

These cysts have been described by a number of surgeons. Some of them have been multilocular, while others were unilocular. Microscopically they are found composed of small cavities or canals, the inner wall of which is lined with an epithelium of the cylindrical, cubic, or polygonal type, while in the larger cysts it is flattened. Malassez believes that these cysts also originate from the paradental epithelial débris, but the reasons why in one case a simple periodontal cyst should develop, while in others cystomata or cystadenomata arise, is as yet insufficiently explained, but a large number of authorities agree that the original epithelial cells of periodontal cysts originate from the enamel organ, while the other types of cysts take their origin from the buccal mucous membrane.

As I have already stated, the development of dentigerous cysts is caused by the increase of the fluid contents, which is evidently constantly secreted by the lining membrane of the cysts, and the development of the growth naturally extends in the direction of least resistance. The bony structures of the jaw enveloping the cyst become thinner and thinner until finally they entirely disappear by pressure atrophy, and then the cyst assumes a soft consistency. If the cyst continues to grow, the soft parts covering it become thin, and finally the cyst ruptures and its fluid contents are evacuated spontaneously. This, however, rarely happens, because operative interference is usually undertaken before a marked development has been reached. I have had one case of this description, which I will here briefly report:

A man aged thirty-two years had complained of an inflammation of the buccal mucosa covering the root of the first molar on the left side about eighteen months before coming under observation, and at the same time he noticed a swelling appearing under the left nostril. A few weeks later the tumor opened spontaneously and a considerable amount of pus escaped. On examination a fistula was found near the root of the left first molar, and the patient stated that the swelling would increase in size and then give exit to quite an amount of pus, after which it would decrease. The pus was always discharged through the fistula. When the tumor would increase in size on account of the collection of pus within it, it gave rise to a certain amount of pain. In other respects the patient was entirely well. The skin over the tumor was movable and not reddened. An incision was made over the tumor, including the fistulous opening, which gave exit to a yellowish fetid pus. The anterior wall of the alveolar process was resected, and then a cavity lined with a membrane was exposed. The cyst sac was carefully dissected out and the cavity packed with gauze. This was removed in three days and the patient was discharged well in eighteen days.

The fluid contents of these cysts are usually serous, and it is only after secondary infection of the cyst has taken place that the liquid becomes purulent. Infection can occur very easily from the buccal cavity or from the remains of teeth within the cyst. In those cases where decayed roots protrude into the cystic cavity, infection may also arise from the canal of the root.

*A*, empyema of sinus; *B*, dentigerous cyst; *C*, dental abscess.

Regarding the diagnosis of dentigerous cysts it may be said that when the growth has reached a certain size but little difficulty will be experienced. We will here find a circumscribed unilateral swelling of the bone giving rise to a parchment crepitation and a fluctuation. If there is any doubt, an exploratory puncture will in most instances give issue to a serous fluid. In the early stages of the process, when the swelling of the bone is of small dimensions and the tumor hard to the feel, the diagnosis is less easy, because in this condition the cyst resembles any circumscribed tumor of the jaw. The absence of pain during the entire progress of the growth will aid one in making a differential diagnosis. Dentigerous cysts might be mistaken for cystomata, but it should be remembered that the latter class of growth pertains almost exclusively to the lower jaw. Suppurating dentigerous cysts may simulate an alveolar abscess, but if the topography of the parts be remembered an erroneous diagnosis can hardly be made, and the accompanying illustration, taken from the excellent "Manuel de Diagnostic chirurgical," by Duplay, shows extremely well the relationship between the various forms of fluid collections occurring in the upper jaw.

If the cyst should burst into the antrum of Highmore, a primary empyema of this cavity will arise, and, *vice versa,* an infected cyst of the superior maxillary opening on the outside might easily simulate an empyema of the antrum if the cystic sac protrudes into the cavity, completely filling it.

The prognosis of dentigerous cysts is in every respect benign. The only serious complication that might arise would be when a cyst of the lower jaw becomes infected, because the danger then would be that the pus might extend and infect secondarily one of the large veins of the neck; but this is only a supposition of my own, for I must confess that I have been unable to find any such instance recorded.

The treatment of these cysts is simple. They should never be punctured unless for diagnostic purposes, and after this, when the diagnosis has been made, operation should be immediately undertaken. In undertaking the surgical cure of these cysts it is absolutely necessary to remove all vestige of the lining membrane, otherwise a relapse is practically certain to arise. In order to do this, the cavity should be freely exposed by a large opening, and the curette, scissors, and, if necessary, the thermo-cautery should be freely used, because if any epithelial cells remain within the cavity they can regenerate and cause recurrence.

After the cavity has been thoroughly cleared of its lining membrane it should be plugged with gauze for a few days, so that the epithelium of the buccal mucous membrane and the granulations arising within the cystic cavity unite, so that the latter becomes filled and only leaves a slight trace behind it.

If any tooth or root is found protruding into the cyst, it should be removed. In large cysts it may be necessary to drain through the floor of the nose, as was done in one or two cases here reported.

When dealing with these cysts developed in the lower jaw, it is oftentimes impossible to reach the tumor by way of the buccal cavity, and it must be attacked through a cutaneous incision. In this case it is indicated to remove as much of the lining membrane of the cyst as possible, and to accomplish this a sufficient resection of the outer shell of bone should be done.

## Figures and Tables

**Figure f1:**